# Response to [Glycated Albumin‐Guided Therapy: Biomarker Utility or Earlier Treatment Intensification?]

**DOI:** 10.1111/1753-0407.70275

**Published:** 2026-10-01

**Authors:** Qian Ren, Linong Ji

**Affiliations:** ^1^ Department of Endocrinology Peking University People's Hospital Beijing People's Republic of China; ^2^ Peking University Diabetes Center Beijing People's Republic of China

Dear Editor,

We thank the authors for their thoughtful comments on our randomized controlled trial of glycated albumin (GA)‐guided therapy in newly diagnosed type 2 diabetes mellitus (T2DM) [1]. We appreciate the opportunity to clarify several key points.

First, we fully agree that the primary endpoint—achievement of HbA1c<7% at 12 weeks—was neutral, and we explicitly reported and discussed this negative result. We had acknowledged that secondary analyses were exploratory and hypothesis‐generating, and we have interpreted them with appropriate caution, as stated in the manuscript.

Second, regarding the HbA1c<6.5% result, we agree that intention‐to‐treat inference is primary. The difference was marginal in the full analysis set (35.7% vs. 23.8%, *p* = 0.065) and nominally significant in the per‐protocol set (40.5% vs. 25.3%, *p* = 0.034). We reported both transparently and did not base our main conclusion on the per‐protocol finding; rather, we view the consistent direction of effect as a signal warranting further investigation.

Third, we agree that the greater improvements in HbA1c reduction, HOMA‐β, HOMA‐IR, and anthropometric parameters in the GA‐guided group may partly reflect earlier and more intensive combination therapy. However, this is precisely the intended clinical utility of GA: to provide a timely, actionable trigger for treatment intensification when HbA1c remains insensitive to short‐term changes. In addition, according to the protocol, in the standard therapy group, apart from withholding the GA measurement data, we did not impose any additional restrictions on blood glucose monitoring; capillary blood glucose testing was permitted as per routine clinical practice. So, the relevant question is not whether GA has intrinsic therapeutic effects, but whether GA measurement facilitates earlier and more effective treatment decisions compared with routine clinical practice. Our trial provides preliminary evidence.

Finally, we agree that short‐term changes in HOMA‐β and HOMA‐IR should not be equated with durable β‐cell recovery or sustained improvement in insulin sensitivity. We interpreted these as short‐term metabolic improvements rather than evidence of long‐term disease modification.

We thank the authors for their insightful comments.

## Author Contributions

Q.R. wrote the manuscript. L.J. reviewed and edited the manuscript. Q.R. and L.J. contributed significantly and in keeping with the latest guidelines of the International Committee of Medical Journal Editors.

## Funding

This work was supported by Asahi Kasei Pharma Corporation and National Clinical Key Specialty Construction Program of China (Department of Endocrinology, Peking University People's Hospital) with support from the central government budget.

## Conflicts of Interest

The authors declare no conflicts of interest.

## Data Availability

Data sharing not applicable to this article as no datasets were generated or analysed during the current study.
